# Susceptibility of Different Mouse Wild Type Strains to Develop Diet-Induced NAFLD/AFLD-Associated Liver Disease

**DOI:** 10.1371/journal.pone.0155163

**Published:** 2016-05-11

**Authors:** Vera H. I. Fengler, Tanja Macheiner, Sonja M. Kessler, Beate Czepukojc, Katja Gemperlein, Rolf Müller, Alexandra K. Kiemer, Christoph Magnes, Johannes Haybaeck, Carolin Lackner, Karine Sargsyan

**Affiliations:** 1 BioPersMed/Biobank Graz, Medical University of Graz, Graz, Austria; 2 Department of Pharmacy, Pharmaceutical Biology, Saarland University, Saarbrücken, Germany; 3 Department of Pharmacy, Pharmaceutical Biotechnology, Saarland University, Saarbrücken, Germany; 4 Department of Microbial Natural Products, Helmholtz-Institute for Pharmaceutical Research Saarland (HIPS), Helmholtz Centre for Infection Research and Pharmaceutical Biotechnology (HZI), Saarbrücken, Germany; 5 Institute for Biomedicine and Health Sciences, Joanneum Research, Graz, Austria; 6 Institute of Pathology, Medical University of Graz, Graz, Austria; RWTH Aachen, GERMANY

## Abstract

Although non-alcoholic and alcoholic fatty liver disease have been intensively studied, concerning pathophysiological mechanisms are still incompletely understood. This may be due to the use of different animal models and resulting model-associated variation. Therefore, this study aimed to compare three frequently used wild type mouse strains in their susceptibility to develop diet-induced features of non-alcoholic/alcoholic fatty liver disease. Fatty liver disease associated clinical, biochemical, and histological features in C57BL/6, CD-1, and 129Sv WT mice were induced by (i) high-fat diet feeding, (ii) ethanol feeding only, and (iii) the combination of high-fat diet and ethanol feeding. Hepatic and subcutaneous adipose lipid profiles were compared in CD-1 and 129Sv mice. Additionally hepatic fatty acid composition was determined in 129Sv mice. In C57BL/6 mice dietary regimens resulted in heterogeneous hepatic responses, ranging from pronounced steatosis and inflammation to a lack of any features of fatty liver disease. Liver-related serum biochemistry showed high deviations within the regimen groups. CD-1 mice did not exhibit significant changes in metabolic and liver markers and developed no significant steatosis or inflammation as a response to dietary regimens. Although 129Sv mice showed no weight gain, this strain achieved most consistent features of fatty liver disease, apparent from concentration alterations of liver-related serum biochemistry as well as moderate steatosis and inflammation as a result of all dietary regimens. Furthermore, the hepatic lipid profile as well as the fatty acid composition of 129Sv mice were considerably altered, upon feeding the different dietary regimens. Accordingly, diet-induced non-alcoholic/alcoholic fatty liver disease is most consistently promoted in 129Sv mice compared to C57BL/6 and CD-1 mice. As a conclusion, this study demonstrates the importance of genetic background of used mouse strains for modeling diet-induced non-alcoholic/alcoholic fatty liver disease.

## Introduction

Non-alcoholic (NAFLD) as well as alcoholic fatty liver disease (AFLD) range among the most prevalent liver diseases and are associated with considerable health and socioeconomic burden in many populations worldwide [1–6]. NAFLD, associated with insulin resistance, represents the hepatic manifestation of the metabolic syndrome (MS) [1, 2, 7, 8], while AFLD is caused by excessive alcohol consumption [5, 6]. Both conditions comprise a spectrum of liver diseases, ranging from steatosis to steatohepatitis and cirrhosis in humans [6, 9–12]. Thereby, the complex pathophysiological mechanisms of NAFLD and AFLD development are influenced by environmental as well as genetic factors [4, 11]. Genetic determinants of NAFLD and AFLD comprise variants of two genes, patatin-like phospholipase domain-containing 3 (PNPLA3) and transmembrane 6 superfamily member 2 (TM6SF2). Single nucleotide polymorphisms (SNPs) in these genes increase hepatic triglyceride (TG) content as well as development of steatosis and fibrosis in humans [13–19]. SNPs more specifically involved in NAFLD development and progression include e.g. variants in insulin receptor substrate-1 (IRS1) and glucose transporter solute carrier family 2 member 1 gene (SLC2A1) [20–22], while AFLD is associated with allelic variations in class I alcohol dehydrogenase (ADH) and aldehyde dehydrogenase (ALDH) [23].

Human NAFLD and AFLD, resulting from over-nutrition, alcohol or the combination of both, can be modelled in wild type (WT) mice by various dietary regimens. High-fat feeding is a common tool to induce liver conditions in mice which are similar to that in human NAFLD. Thereby, different formulations of high-fat diets with varying relative carbohydrate and fat content ranging from 35% to 70% of total calories are published. Moreover, high-fat diets with differences in the saturation of the nutritional fatty acids are used [24–35]. Nevertheless, a comparison of high-fat diets with variations in the saturation index of the fat resulted in almost similar development of steatosis and hepatic inflammation [36]. A further difference in published high-fat diets comprises nutritional cholesterol content, which seems to have a crucial impact on the development of hepatic inflammation in mice [36, 37]. Also carbohydrate type and content of the used diets are influencing development of steatosis. Especially diets enriched in fructose induce obesity, steatosis, inflammation and metabolic changes in mice [38–43]. Western type called diets combine high-fat, increased carbohydrate and high-cholesterol content and induce steatosis, hepatic inflammation, and at late stage fibrogenesis also in mice [29, 43–47]. Alternatively, conditions of hepatic steatosis, inflammation, and liver fibrosis can be modeled by feeding diets deficient in methionine and/or choline (MCD), however these diets do not cause NAFLD in the context of the MS, e.g. they cause weight loss [24, 48].

Traditionally, for modeling diet-induced NAFLD and AFLD male C57BL/6, CD-1, and 129Sv WT mouse strains are among the most frequently used [26–28, 30, 34, 47]. Thereby, gender and genetic determinants of the mice may affect their susceptibility to develop NAFLD/AFLD. Gender specific differences in the development of NAFLD in mice have been reported. Male rodents exhibit an increased susceptibility to develop NAFLD, which is opposed to observations in humans, where women seem to have a higher prevalence for NAFLD [9, 49–51]. Known genetic determinants of C57/BL/6 mice affecting development of NAFLD comprise a higher sterol regulatory element-binding protein-1c (SREBP-1) and stearoyl-coenzyme A desaturase 1 (SCD-1) expression and activity [27]. Additionally, C57BL/6 mice seem to be more prone to a NAFLD-like phenotype, through mechanisms involving macrophage activation [52].

Although the genetic background is expected to alter the manifestation of NAFLD and AFLD in different mouse strains this issue has not been sufficiently addressed in literature. Thus, this study aimed to compare the susceptibility of C57BL/6, CD-1, and 129Sv WT mice to develop diet-induced features of NAFLD and AFLD. Mice of each strain were divided into four groups receiving (i) liquid Lieber DeCarli high-fat diet (HF), (ii) liquid Lieber DeCarli diet supplemented with ethanol (EtOH), (iii) liquid Lieber DeCarli high-fat diet supplemented with ethanol (HF + EtOH) and (iv) an untreated group [25, 35, 48, 53]. Metabolic markers, liver-related serum biochemistry, liver histology and hepatic inflammation of all mice were compared. To further characterize hepatic response to different dietary regimens, hepatic lipid as well as fatty acid profiles were determined in 129Sv mice. As expected, metabolic parameters as well as hepatic response to dietary regimens differed to a great extent between the three WT mouse strains. Reported in literature and also observed here, C57BL/6 mice exhibited high deviations within the development of steatosis and inflammation [28, 30, 34]. Surprisingly, CD-1 mice did not show significant features of fatty liver disease as a response to the dietary regimens. Most consistently, 129Sv mice developed features of NAFLD/AFLD, with considerably altered hepatic lipid and fatty acid profiles as a result of dietary regimens. Thus, this study is demonstrating that WT mouse strains differ in their susceptibility to develop a diet-induced NAFLD/AFLD-like phenotype and further reveals the influence of genetic background on mouse models of diet-induced NAFLD and AFLD.

## Materials and Methods

### Mice and diets

The animal protocol (BMWF-66.010/0081-II/3b/2012) has been approved by the Animal Welfare Committee of the Medical University of Graz and the Austrian Federal Ministry of Science and Research Ref. II/3b. Housing of mice was conducted with food and water ad libitum and monitored in accordance with the Animal Welfare Policy of the Medical University of Graz.

Male C57BL/6N (C57BL/6), 129S2/Sv (129Sv), and CD-1 mice (Charles River Laboratories, Germany) were group-housed in individually ventilated cages in a specific pathogen free (SPF) facility with a 12 hour light and dark cycle. After an acclimatization period, the 8 to 10 weeks old mice were weight matched and divided into four groups (n = 5). The first group received liquid Lieber DeCarli high-fat diet (HF) (46.23% fat: 28.17% corn oil, 16.49% olive oil, 1.57% safflower oil) (Ssniff, Germany). The second group was treated with ethanol by feeding liquid Lieber DeCarli diet (EtOH) and the third group received liquid Lieber DeCarli high-fat diet supplemented with ethanol (HF + EtOH) [25]. Mice were adapted to liquid diet before ethanol concentration was increased by 1% every third day to finally 5% for EtOH and 2.5% for HF + EtOH. Mice received HF for 7 weeks, EtOH for 12, 14, and 16 weeks, and HF + EtOH for 5, 7, and 9 weeks, respectively. The fourth group was sacrificed after the acclimatization period and served as untreated group. To collect tissue and blood samples, mice were anesthetized by isoflurane inhalation and decapitated. Tissue samples were snap frozen and stored in liquid nitrogen. An additional liver aliquot was fixed in 10% formalin.

### Biochemical analyses of serum parameters

Serum was collected by centrifugation (5,000 g, 15 min, room temperature). Aspartate aminotransferase (AST), alanine aminotransferase (ALT), serum TG, serum cholesterol (Roche Diagnostics, Switzerland) and serum free fatty acids (FFA) (Wako Chemicals GmbH, Japan) were determined by using commercial assay kits according to the manufacturer’s instructions.

### Histological analysis

After formalin fixation, livers were embedded in paraffin and sectioned at 3 μm. All slices were stained with hematoxylin and eosin (HE) for microscopic examination, which was conducted by two board certified pathologists (J.H., C.L.) in a blinded study setup. Additionally, snap frozen liver specimens were sectioned at 6 μm and stained with Oil Red O to illustrate hepatic lipid accumulation.

To quantitatively assess the hepatic effects caused by the different dietary regimens steatosis, inflammation, and fibrosis were scored, analogous to the NAFLD activity score (NAS) from the NASH-CRN [10].

### Immunoassay of hepatic inflammation parameters

Cryopreserved liver specimens were homogenized, supernatants were centrifuged twice (16,000 g, 10 min, 4°C) and afterwards total protein concentration was measured with *DC* Protein Assay Kit (Bio-Rad, USA). Interleukin 6 (IL-6), tumor necrosis factor α (TNFα), and hepatic monocyte chemo attractant protein-1 (MCP-1) contents were measured with a ProcartaPlex^TM^ Immunoassay (eBioscience, USA) according to the manufacturer’s instructions. Thus, supernatants of liver homogenates were diluted to 10 mg/ml total protein concentration and 25 μl of this dilution were used for measurements.

### Analysis of lipid classes

Lipid extraction and thin layer chromatography (TLC) analysis was performed as previously described using a sulfuric acid/ethanol mixture for detection [54]. In short, 15 mg of the freeze-dried tissue was dispersed in hexane/2-propanol [3:2 (v/v)] for 10 min and centrifuged at 4°C, 10,000 g for 10 min. The nitrogen stream-dried supernatant was dissolved in chloroform/methanol [1:1 (v/v)] and applied onto the TLC plates, which were prewashed with a mixture of chloroform/methanol [2:1 (v/v)] and activated at 110°C for 1 h. TLC plates loaded with samples and standard substances were first developed up to the mid of the plates in chloroform/methanol/acetic acid/water [50:30:8:3 (v/v/v/v)] and then fully developed in heptane/diethyl ether/acetic acid [70:30:2 (v/v/v)]. The ratio area density of each band was quantified using the ImageJ software.

### Fatty acid profile analysis by GC-MS

Cholesterol and fatty acids (FA) of snap-frozen and lyophilized liver tissue samples were extracted using the fatty acid methyl ester (FAME) method and measured with GC-MS as previously published [55].

### Statistical analyses

All results are expressed as medians with interquartile range if not stated otherwise. For data analyses Kruskal-Wallis followed by Dunns test of selected pairs of columns or Mann-Whitney U test were used. Differences were considered significant for *P* values of <0.05. Sample size was calculated by assuming the effect size and the variance for NAS of untreated and high-fat diet fed mice based on literature data. The calculation for an experiment with eight treatment groups with a power of 0.2 and a significance level of 0.05 requires 5 mice per group.

## Results

### Effects of high-fat and ethanol on body parameters

**Fig 1:**
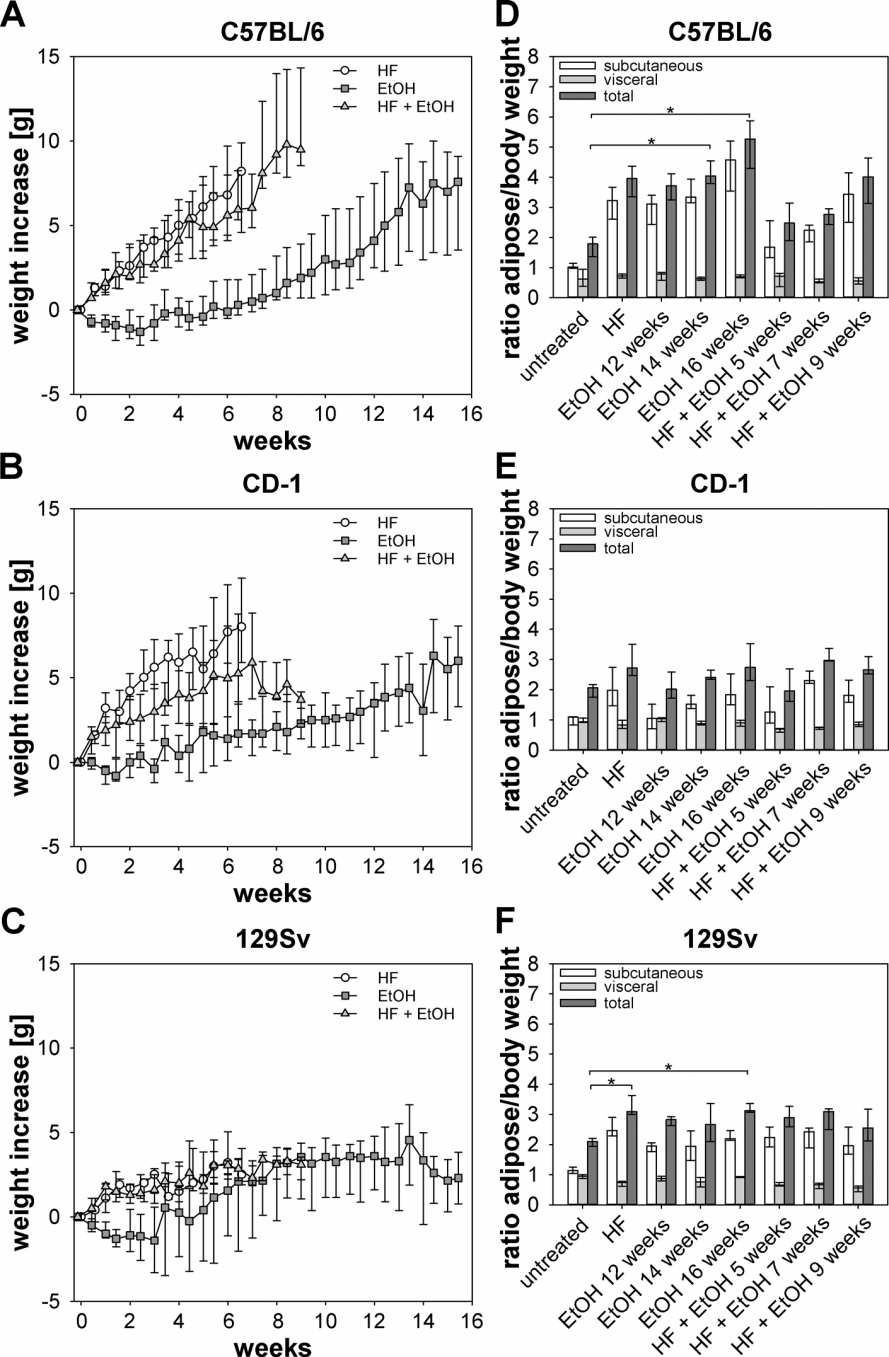
Weight increase and adipose to body weight ratio of different dietary groups and mouse strains. (A-C) Weight increase of HF, EtOH, and HF + EtOH groups and different mouse strains was monitored twice a week, resulting in regimen induced weight increase in C57BL/6 and CD-1 but not in 129Sv mice. (D-F) Adipose to body weight ratios of respective strains and dietary groups, showing increased subcutaneous as well as total adipose to body weight ratio in C57BL/6 and 129Sv mice, while CD-1 mice exhibited only minor changes due to HF, EtOH, and HF + EtOH feeding. At the end of each experiment, subcutaneous as well as visceral adipose tissue was dissected, weighted and subcutaneous adipose, visceral adipose, and total adipose to body weight ratios were calculated. Shown are medians with interquartile ranges and relevant significant differences are marked by an asterisk (Kruskal-Wallis followed by Dunns test of selected pairs of columns, *P* values of <0.05).

To compare the effects of dietary regimens on body weight, C57BL/6, CD-1, and 129Sv mice were fed with the respective diets as described in Material and Methods. Weight increase was monitored twice a week and at the end of each experiment subcutaneous and visceral fat pads were weighted to determine fat to body weight ratio. For C57BL/6 as well as CD-1 mice, body weight increase of the groups fed HF and HF + EtOH was significantly increased compared to mice fed EtOH only at time point 7 weeks (Fig 1A and 1B). 129Sv mice showed no significant differences in weight gain between the dietary groups (Fig 1C). Comparison of the different strains resulted in a significantly higher weight increase of C57BL/6 mice in comparison to CD-1 and 129Sv mice after 9 weeks of feeding HF + EtOH (Fig 1A–1C). Only four out of five 129Sv mice of each feeding duration survived the end of EtOH feeding experiment, therefore only four mice of each time point were available for sample and data acquisition.

Total adipose to body weight ratios of C57BL/6 mice showed an increase of all dietary groups compared to the untreated group, whereas ratios of subcutaneous and visceral adipose to body weight after 14 and 16 weeks EtOH feeding were significantly elevated compared to untreated mice. Furthermore, total adipose to body weight ratios of EtOH and HF + EtOH fed C57BL/6 mice ascended from the earliest to the latest time point (Fig 1D). For CD-1 mice only a slight tendency towards elevated total adipose to body weight ratio of dietary groups compared to the untreated group was observed (Fig 1E). In 129Sv mice HF and also EtOH feeding for 16 weeks caused a significant increase in total adipose to body weight ratios compared to untreated mice (Fig 1F). All three mouse strains exhibited an equal distribution of visceral and subcutaneous adipose tissue prior to the dietary treatments. Due to HF and EtOH feeding the amount of subcutaneous adipose tissue increased, while visceral adipose mass was not altered (Fig 1D–1F).

Liver to body weight ratios of all three mouse strains did not show significant differences between dietary groups (data not shown).

### Liver markers and metabolic serum parameters upon high-fat and ethanol exposure

**Fig 2:**
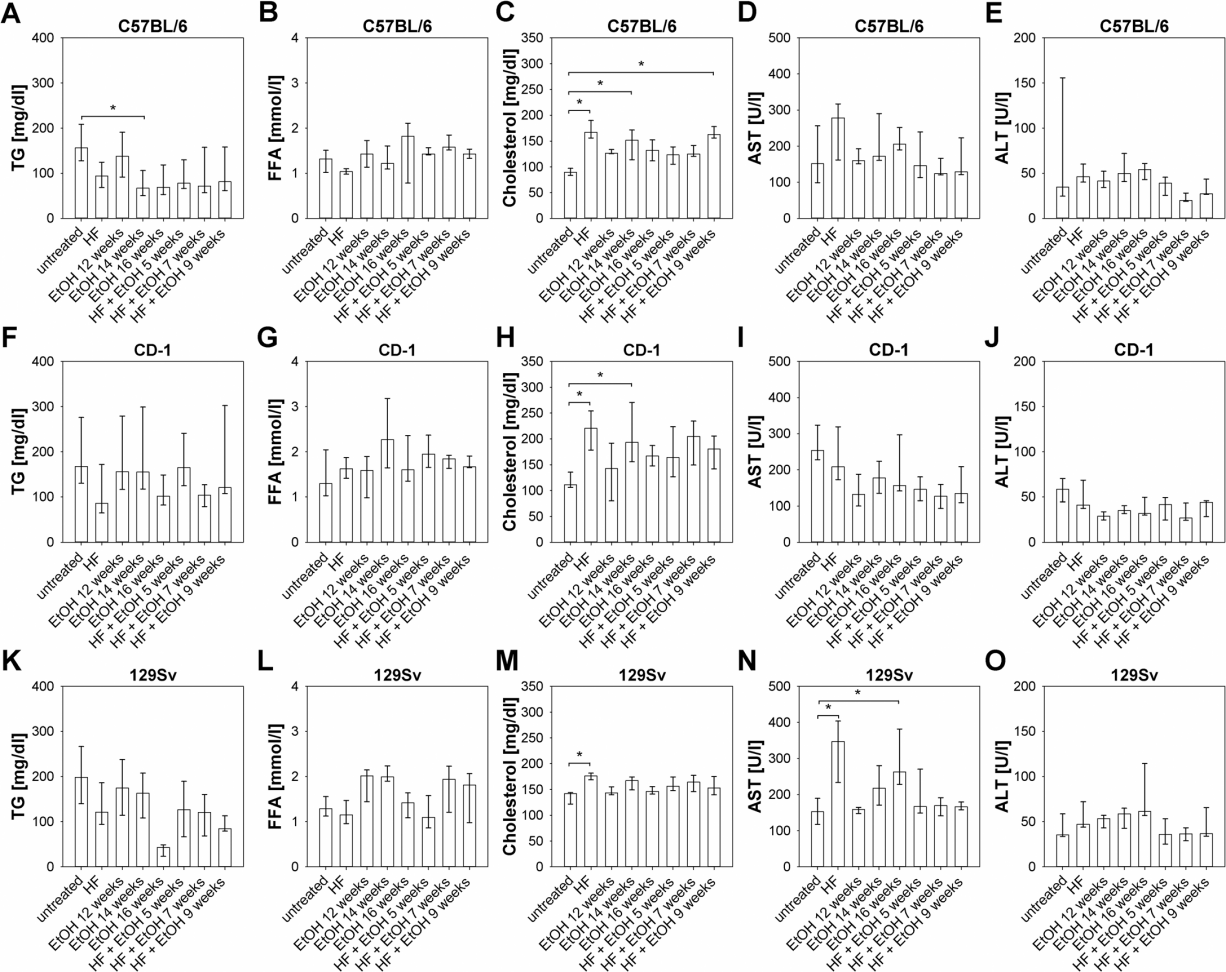
Serum levels of TG, total cholesterol, FFA and liver function, transaminases AST and ALT of all regimen groups of C57BL/6, CD-1, and 129Sv mice. (A-E) Metabolic serum parameters and transaminase levels of C57BL/6 mice showed significant increases in total serum cholesterol of HF, EtOH 14 weeks, and HF + EtOH 9 weeks fed mice and a decrease of serum TG in mice fed with EtOH for 12 weeks compared to untreated mice. (F-J) Alterations in serum metabolic and hepatic parameters of regimen treated CD-1 mice led to significant elevated total cholesterol levels in mice fed with HF and EtOH for 14 weeks compared to untreated counterparts. (K-O) Serum concentrations of metabolic and hepatic parameters in 129Sv mice showed increased cholesterol level in the HF group and elevated AST in HF and EtOH 16 weeks fed mice compared to the untreated group. Shown are medians with interquartile ranges and relevant significant differences are marked by an asterisk (Kruskal-Wallis followed by Dunns test of selected pairs of columns, *P* values of <0.05).

General as well as hepatic metabolic changes induced by HF, EtOH, and HF + EtOH feeding were at first determined by serum concentrations of TG, FFA, cholesterol as well as transaminases AST and ALT. Dietary regimens caused clearance of serum TG in all three strains with significantly lower TG levels in C57BL/6 fed with EtOH for 14 weeks compared to untreated mice and no accumulation of FFA in all mouse strains (Fig 2A, 2B, 2F, 2G, 2K and 2L). Total serum cholesterol, in contrast, was elevated in all dietary groups compared to the untreated group, whereas differences between untreated groups and HF fed groups were significant in all mouse strains (Fig 2C, 2H and 2M).

In C57BL/6 and 129Sv mice HF and EtOH feeding led to an increase in serum AST and ALT levels. However, these only reached statistical significance in 129Sv mice fed with HF and EtOH for 16 weeks. Feeding HF + EtOH led to similar AST and ALT levels compared to the untreated group for C57BL/6 as well as 129Sv mice (Fig 2D, 2E, 2N and 2O). Surprisingly, CD-1 mice displayed lower AST as well as ALT levels in all dietary groups compared to the untreated mice (Fig 2I and 2J).

### Induction of hepatic steatosis, ballooning, and inflammation by high-fat and ethanol feeding

**Fig 3:**
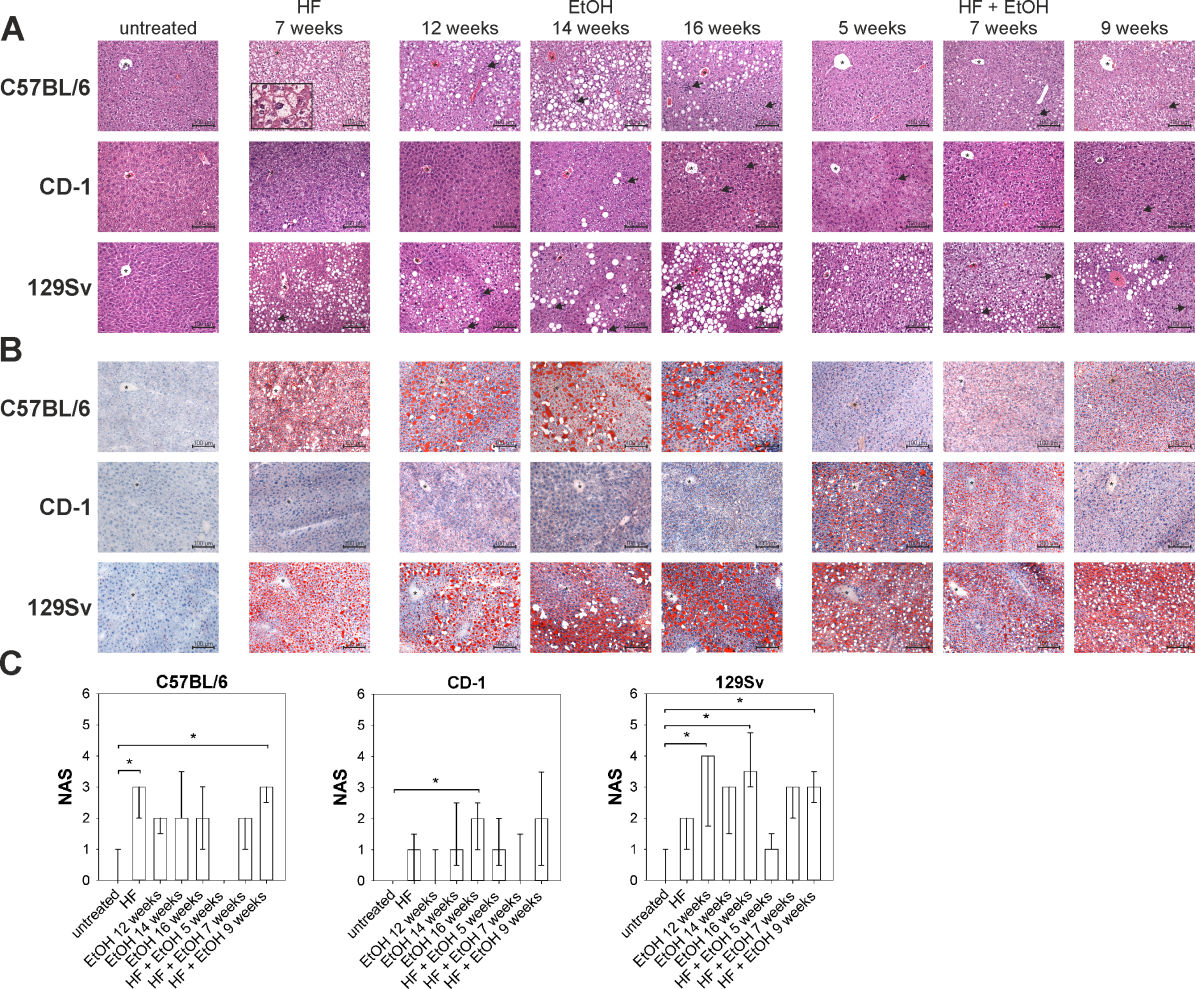
Liver histology visualized by HE and Oil Red O staining as well as NAS quantification of dietary induced liver disease. (A) Mouse livers of different dietary groups and mouse strains were HE stained to show intracellular lipid accumulation, ballooning, and inflammation, with increases in hepatic lipid content and inflammation in HF and EtOH fed C57BL/6 and 129Sv mice. Shown are representative pictures of each group (magnification 200). Asterisks in each picture mark a central vein and black arrows indicate inflammatory cell infiltration. The enclosed box in C57BL/6 HF diet 7 weeks shows an enlarged image of cells resembling ballooning (magnification 600). (B) Oil Red O stained liver sections of regimen treated mouse strains, showing prominent lipid accumulation in regimen treated C57BL/6 and 129Sv mice, but none in CD-1 mice. Depicted are representative sections of each group (magnification 200). Asterisks in each picture mark a central vein. (C) Quantification of diet-induced steatosis, ballooning, and inflammation by NAS-scoring. NAS was significantly increased in C57BL/6 mice fed with HF and HF + EtOH 9 weeks compared to their untreated counterparts, while in CD-1 mice only EtOH fed for 16 weeks increased NAS. Feeding EtOH for 12 as well as 16 weeks and HF + EtOH for 9 weeks elevated NAS in 129Sv mice significantly compared to untreated mice. Given are medians with interquartile ranges and relevant significant differences are marked by an asterisk (Kruskal-Wallis followed by Dunns test of selected pairs of columns, *P* values of <0.05).

Main histological features of NAFLD/AFLD were investigated by microscopic examination of HE and Oil Red O stained liver sections. To further quantitatively assess effects of the different dietary regimens on the development of steatosis and steatohepatitis within the three different mouse strains, the NAS was used, as described in Materials and Methods [10]. Livers of untreated mice of all three strains appeared histologically normal without steatosis, ballooning or inflammation, except for a few livers showing few infiltrating lymphocytes (Fig 3 and Table 1).

**Table 1:** Medians and interquartile ranges of histopathological features of steatosis, ballooning, and inflammation as well as NAS of regimen treated C57BL/6, CD-1, and 129Sv mice.

Strain	Factor	untreated	HF	EtOH			HF + EtOH		
			7 weeks	12 weeks	14 weeks	16 weeks	5 weeks	7 weeks	9 weeks
**C57BL/6**	**Steatosis**	0 (0–0)	1 (0–1)	1 (0.5–1)	1* (1–1)	1 (0–1)	0 (0–0)	1 (0–1)	0 (0–1)
	**Ballooning**	0 (0–0)	2* (1.5–2)	0 (0–0)	0 (0–0)	0 (0–0)	0 (0–0)	0 (0–0.5)	2 (0.5–2)
	**Inflammation**	0 (0–1)	0 (0–0.5)	1 (0.5–1.5)	1 (1–2.5)	2 (0.5–2)	0 (0–0)	1 (0.5–1)	1 (0–2)
	**NAS**	0 (0–1)	3* (2–3)	2 (1.5–2)	2 (2–3.5)	2 (1–3)	0 (0–0)	2 (1–2)	3* (2.5–3)
**CD-1**	**Steatosis**	0 (0–0)	0 (0–0.5)	0 (0–0)	1 (0–1)	0 (0–0.5)	0 (0–0.5)	0 (0–0)	0 (0–0)
	**Ballooning**	0 (0–0)	0 (0–0)	0 (0–0)	0 (0–0)	0 (0–0)	0 (0–0)	0 (0–0)	1 (0–1.5)
	**Inflammation**	0 (0–0)	0 (0–1.5)	0 (0–1)	1 (0–1.5)	2 (1–2)	1 (0.5–1.5)	0 (0–1.5)	1 (0.5–2)
	**NAS**	0 (0–0)	1 (0–1.5)	0 (0–1)	1 (0.5–2.5)	2* (1–2.5)	1 (0.5–2)	0 (0–1.5)	2 (0.5–3.5)
**129Sv**	**Steatosis**	0 (0–0)	1 (1–1)	1 (1–1)	1 (1–1)	1* (1–1.8)	1 (0.5–1)	1 (0.5–1)	1 (0.5–1)
	**Ballooning**	0 (0–0)	0 (0–0)	0 (0–0)	0 (0–0)	0 (0–0)	0 (0–0)	0 (0–0)	0 (0–0)
	**Inflammation**	0 (0–1)	1 (0–1)	3 (0.75–3)	2.(0.5–2)	2.5* (2–3)	0 (0–1)	2 (1.5–2)	2 (2–2.5)
	**NAS**	0 (0–1)	2 (1–2)	4* (1.75–4)	3 (1.5–3)	3.5* (3–4.75)	1 (1–1.5)	3 (2–3)	3* (2.5–3.5)

* differences to untreated group were considered significant for *P* values of <0.05 by Kruskal-Wallis followed by Dunns test of selected pairs of columns

Histopathological examination of C57BL/6 livers revealed microvesicular and mild macrovesicular steatosis in HF and HF + EtOH fed mice. Cells resembling ballooning were observed upon HF feeding and additionally in livers of C57BL/6 mice fed with HF + EtOH for 9 weeks and both dietary groups exhibited a significant higher NAS compared to the untreated group. EtOH fed groups showed increased steatosis and inflammation, but no ballooning (Fig 3 and Table 1).

In CD-1 mice the dietary regimens resulted in minor features of NAFLD/AFLD, as nearly no steatosis was observed. Ballooning did also not occur and inflammatory cells were rare. Compared to the untreated group, the NAS was only significantly increased in the group fed with EtOH for 16 weeks, whereas this was mainly due to increased inflammation (Fig 3 and Table 1).

In contrast, 129Sv livers exhibited moderate steatosis, present in nearly all livers obtained from the three different dietary groups. However, ballooning was not shown in any of the livers from 129Sv mice. Inflammatory cell infiltration was prominently noted in EtOH and HF + EtOH fed groups and ascended from the shortest to the longest feeding duration (Fig 3 and Table 1).

Additionally, in none of the untreated or regimen treated mice of either strain fibrosis was observed.

The statistical correlation of NAS and dietary regimens compared in C57BL/6, CD-1, and 129Sv mice is shown in S1 Table (S1 Table).

### Inflammatory response in livers of high-fat and ethanol fed mice

**Fig 4:**
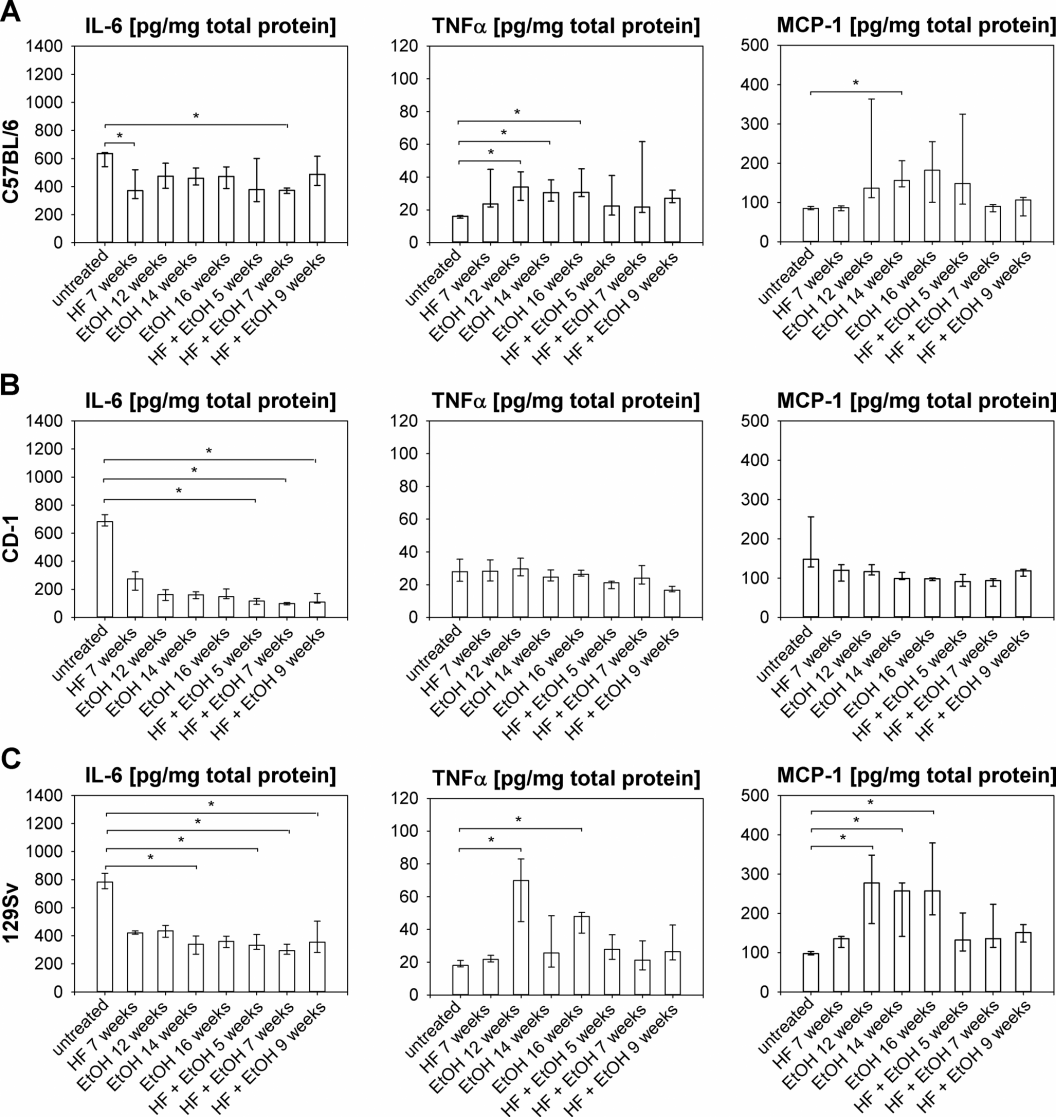
Measurement of inflammation markers IL-6, TNFα, and MCP-1. Inflammation markers were determined out of liver homogenates of all livers of the respective mouse strains and dietary groups. IL-6, TNFα, and MCP-1 levels of (A) C57BL/6 mice, (B) CD-1 mice, and (C) 129Sv mice, indicating inflammatory response in C57BL/6 and 129Sv mice by decreased hepatic IL-6 and increased hepatic TNFα and MCP-1 concentrations due to regimen feeding. Given are medians with interquartile ranges and relevant significant differences are marked by an asterisk (Kruskal-Wallis followed by Dunns test of selected pairs of columns, *P* values of <0.05).

To quantify inflammation in livers of dietary regimen treated mice, interleukin-6 (IL-6), tumor necrosis factor alpha (TNFα) and monocyte chemoattractant protein-1 (MCP-1) were measured in liver tissue and results are shown in Fig 4. The IL-6 level in untreated C57BL/6 mice was at least 1.3 times higher than in mice of the different dietary groups, which is consistent with previous reports, in which elevated hepatic IL-6 levels corresponded with healthy livers, in contrast to increased systemic IL-6, which is associated with NAFLD/AFLD [48, 56–58]. 7 weeks of HF and HF + EtOH feeding significantly decreased IL-6 level of C57BL/6 mice compared to untreated mice (Fig 4A). TNFα possesses a key role in steatohepatitis development by enhancing fibrosis [57, 58] and hepatic TNFα was significantly increased in EtOH fed C57BL/6 mice compared to untreated mice (Fig 4A). MCP-1 is important for monocyte/macrophage recruitment in the liver and is thought to initiate inflammation in NAFDL/AFLD [32, 59]. EtOH feeding for 14 weeks elevated hepatic MCP-1 in C57BL/6 mice significantly compared to no treatment (Fig 4A).

Untreated CD-1 mice exhibited high hepatic IL-6 concentrations, whereas HF + EtOH caused significantly lower IL-6 values. TNFα and also MCP-1 were similar, or sligthly decreased in the dietary groups compared to untreated mice (Fig 4B).

In 129Sv mice a decrease in hepatic IL-6 was observed as a result of all dietary regimens. Thereby, differences between untreated mice and the EtOH group, at time point 14 weeks and at all time points of HF + EtOH group were significant. TNFα levels of 129Sv mice significantly elevated due to EtOH feeding in mice fed for 12 and 16 weeks compared to untreated mice. All dietary regimens led to elevated hepatic MCP-1 levels compared to the untreated group, whereat, significant differences were observed for all time points of EtOH feeding (Fig 4C).

### Hepatic lipid profile of regimen treated CD-1 and 129Sv mice

**Fig 5:**
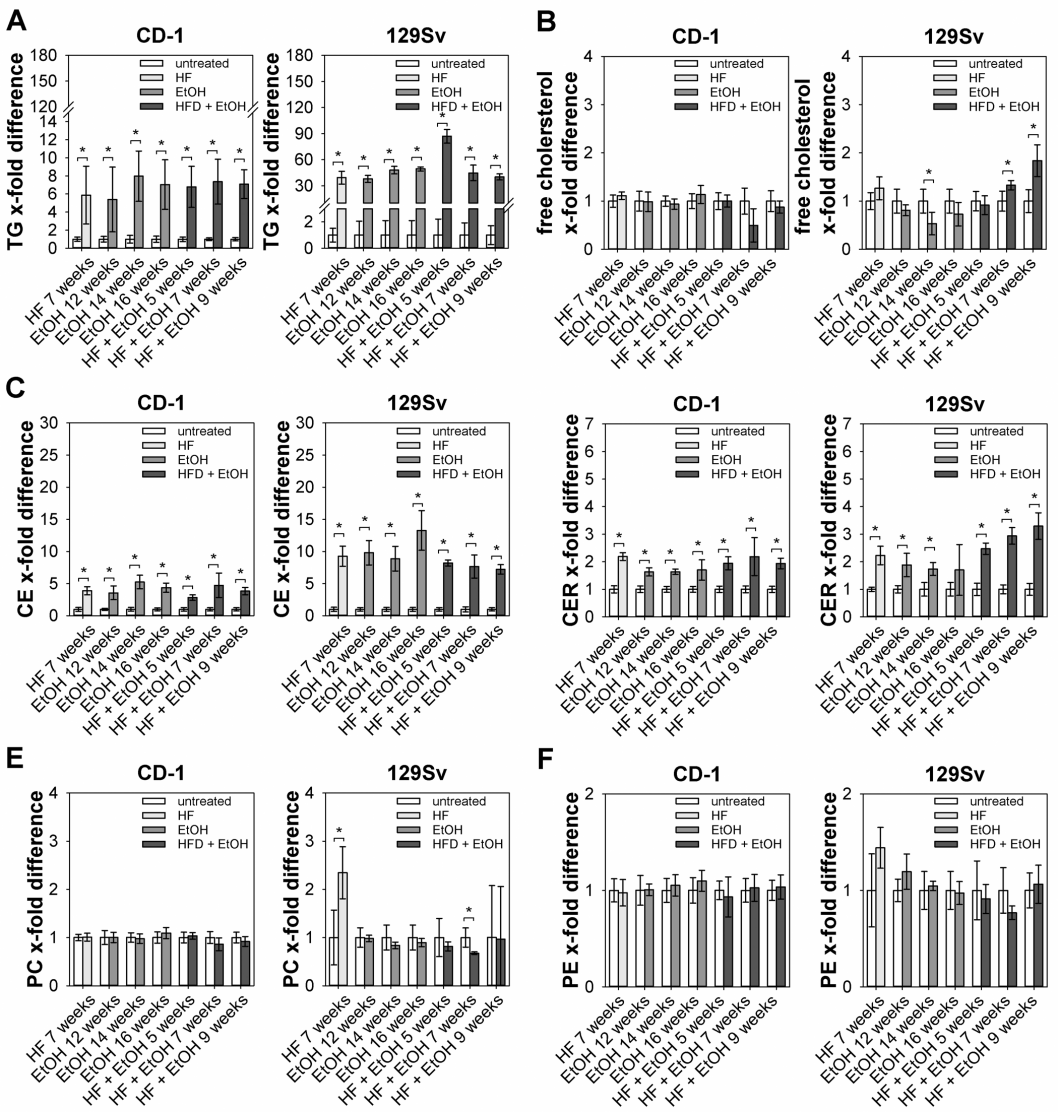
Determination of hepatic lipid profile of regimen treated CD-1 and 129Sv mice. Hepatic lipid content was determined out of livers from regimen fed CD-1 and 129Sv mice by a semi-quantitative chromatographic method and is shown as relative amount compared to untreated counterparts. (A) TG levels were increased in both mouse strains, with elevated hepatic TG storage in 129Sv mice compared to CD-1 mice. (B) Free cholesterol was only altered in some of the regimen treated 129Sv mice, including decreased free cholesterol in feeding group EtOH 14 weeks and increased free cholesterol in HF + EtOH fed for 7 and 9 weeks. (C) CE as well as (D) CER levels were increased in all feeding groups of either strain, with higher CE content in 129Sv mice compared to CD-1 mice. (E) PC levels were not altered in CD-1 mice, while HF feeding for 7 weeks caused elevated PC content and HF + EtOH feeding for 7 weeks caused decreased PC levels. (F) PE content was similar in both strains, with no alterations between untreated and regimen treated mice. Values are given as means with standard deviations and relevant significant differences are marked by an asterisk (Mann-Whitney U test, *P* values of <0.05).

Due to stable response of 129Sv mice to dietary regimens the metabolic status of these mice was further characterized by determining hepatic lipid profiles. In addition, hepatic lipid composition of CD-1 mice was determined to elucidate differences in lipid accumulation between the two strains. TG levels of CD-1 and 129Sv mice were significantly elevated in all dietary groups compared to the untreated group, whereat TG storage was increased in 129Sv mice compared to CD-1 mice (Fig 5A). Free hepatic cholesterol was significantly increased only in 129Sv mice fed with HF + EtOH for 7 and 9 weeks, but was significantly decreased in 14 weeks of EtOH feeding group (Fig 5B), while it was not altered in CD-1 mice. Hepatic cholesterol ester (CE) levels were significantly elevated in all dietary groups compared to untreated mice, in CD-1 as well as 129Sv mice. Also increase in hepatic CE content was higher in 129Sv mice compared to CD-1 mice (Fig 5C). Hepatic ceramide (CER) levels were significantly higher in both, dietary regimen treated CD-1 and 129Sv mice (Fig 5D). Phosphatidylcholine (PC) levels were only significantly increased in HF fed 129Sv mice compared to untreated mice (Fig 5E), while no differences were observed in CD-1 mice. Phosphatidylethanolamine (PE) levels were not affected by the dietary regimens, neither in CD-1 nor in 129Sv mice (Fig 5F).

Furthermore, lipid composition of subcutaneous fat pads of untreated and HF fed 129Sv and CD-1 mice was compared to elucidate differences in extrahepatic lipid accumulation between the two strains. Subcutaneous adipose of both strains mostly consisted of TG, whereat an increased TG storage of HF fed 129Sv mice compared to untreated mice was observed (data not shown).

### Changes in hepatic fatty acid composition of 129Sv mice upon regimen treatment

**Fig 6:**
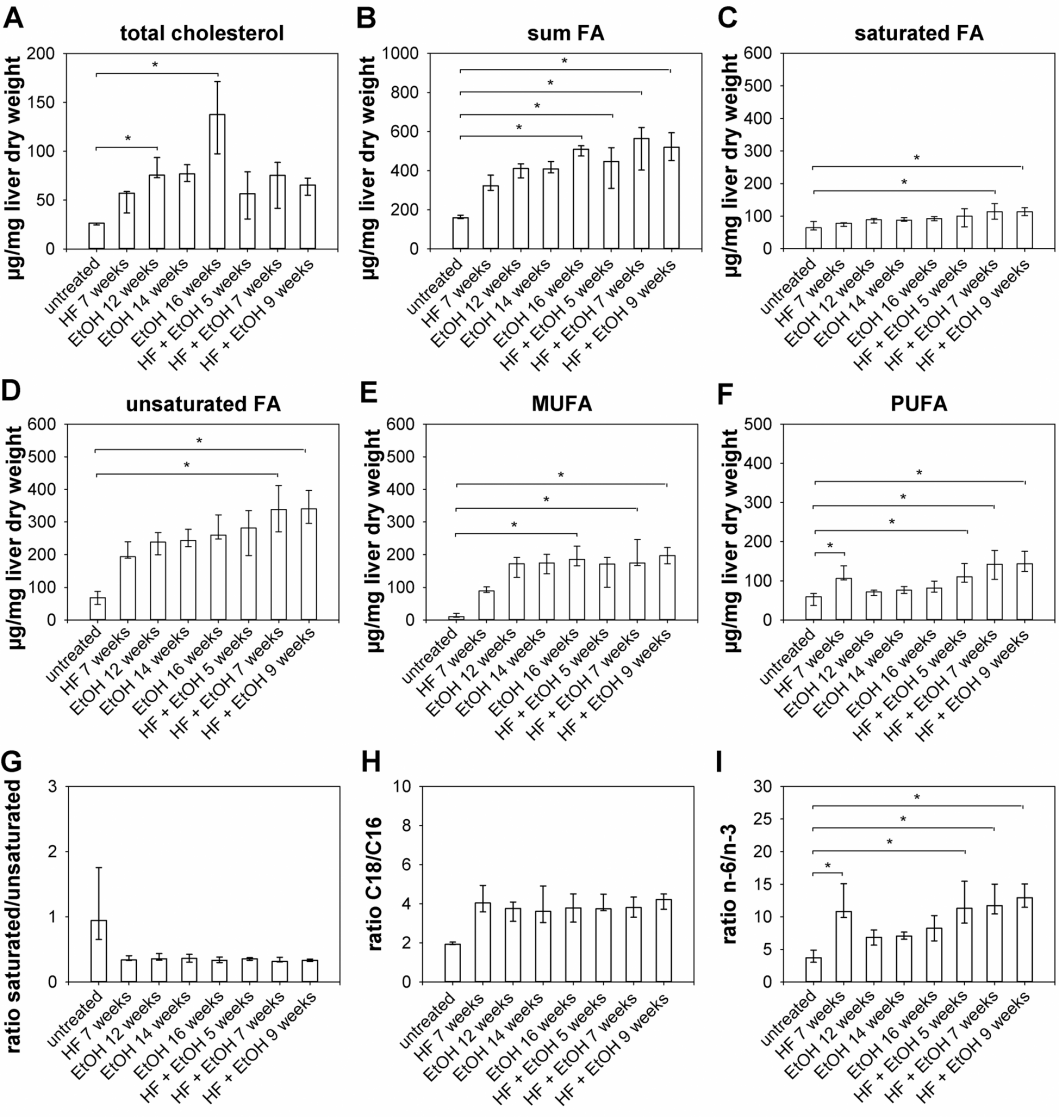
FA composition of regimen treated 129Sv mice livers. FA were determined out of livers from regimen fed 129Sv mice by GC-MS. (A) Total hepatic cholesterol was significantly increased by feeding EtOH for 12 and 16 weeks. (B) Sum of FA was elevated in mice fed with EtOH for 16 weeks as well as in mice fed with HF + EtOH for 7 and 9 weeks. (C) Saturated FA and (D) unsaturated FA were increased in 129Sv mice fed with EtOH for 14 and 16 weeks. (E) MUFA elevated due to feeding EtOH for 16 weeks and HF + EtOH for 7 and 9 weeks respectively. (F) PUFA were only increased in 129Sv mice either receiving HF or HF + EtOH. (G) Ratios of saturated to unsaturated and (H) C18 to C16 FA were not affected by dietary regimens, while (I) ratio of n-6 to n-3 FA was increased due to HF and HF + EtOH feeding at all time points. Shown are medians with interquartile ranges and relevant significant differences are marked by an asterisk (Kruskal-Wallis followed by Dunns test of selected pairs of columns, *P* values of <0.05).

To further characterize changes in hepatic metabolism of regimen treated 129Sv mice FA analysis by GC-MS was performed, resulting in significantly higher total hepatic cholesterol amounts in 129Sv mice fed with EtOH for 12 weeks and 16 weeks, while in all other dietary groups total cholesterol was slightly increased (Fig 6A). An increase of total FA was observed in all dietary groups, but reached statistical significance only in livers of 129Sv mice fed with EtOH for 16 weeks as well as in livers of mice fed with HF + EtOH for 7 and 9 weeks compared to untreated mice, respectively (Fig 6B). Regarding the composition of FA saturated FA only increased slightly in dietary groups, with significantly increased amounts in HF + EtOH fed mice treated for 7 and 9 weeks, respectively (Fig 6C). Unsaturated hepatic FA were elevated in all dietary groups, but reached statistical significance also only in groups fed HF + EtOH for 7 and 9 weeks (Fig 6D). Monounsaturated fatty acids (MUFA) were increased in all dietary groups, with significant increases in groups fed with EtOH for 16 weeks as well as in groups treated with HF + EtOH for 7 and 9 weeks, respectively (Fig 5E). In contrast, polyunsaturated fatty acids (PUFA) were not affected by EtOH feeding and were only increased in HF fed 129Sv mice and in HF + EtOH fed mice for 5 and 9 weeks (Fig 6F). The ratio of saturated to unsaturated FA was decreased in all dietary groups. However, these differences reached no statistical significance (Fig 6G). Ratio of C18 to C16 FA was increased in all dietary regimen fed 129Sv mice, but also did not reached statistical significance (Fig 6H). In contrast, ratio of n-3 to n-6 FA was increased in all dietary groups, with significant increased values in livers of HF fed 129Sv mice as well as in all mice fed with HF + EtOH (Fig 6I). In all 129Sv mice palmitic acid (C16:0) and stearic acid (C18:0) were the predominant unsaturated FA, which were only significantly increased by feeding HF + EtOH. The predominant MUFA in all 129Sv mice was oleic acid (C18:1ω9cis and C18:1ω9cis), which was also significantly elevated upon HF + EtOH feeding. Linoleic acid (C18:2ω6,9allcis), arachidonic acid (C20:4 ω6,9,12,15cisall), and docosahexanoic acid (C22:6 ω3,6,9,12,15,18allcis) represented the predominant PUFA, whereat linoleic acid (C18:2ω6,9allcis) was specifically increased due to HF feeding and arachidonic acid (C20:4 ω6,9,12,15cisall) was increased due to all dietary regimens. In contrast, docosahexanoic acid (C22:6 ω3,6,9,12,15,18allcis) was decreased due to all dietary regimens (S2 Table).

## Discussion

The herein used liquid dietary regimen, reported by Lieber et al., induces steatosis and inflammation in rats after only 3 weeks of treatment and, at the same, avoids nutritional deficiencies occurring during MCD feeding and alike regimens [25]. In this study, C57BL/6 mice fed HF and EtOH exhibited obesity, apparent from elevated weight gain and increased adipose to body weight ratios. HF as well as EtOH feeding led to metabolic abnormalities including decreased circulating TG levels, most probably due to induction of lipoprotein lipase and subsequent elevated TG clearance [60]. Furthermore, hypercholesterolemia was observed in all dietary groups. Hepatic reaction of C57BL/6 mice to the dietary regimens was heterogeneous. Some mice of the HF group developed several biochemical and histopathological key features of NAFLD/AFLD, such as steatosis, inflammation, and enlarged hepatocytes resembling ballooning, while other mice featured no liver disease at all. Also in the other regimen groups some mice showed diseased livers, while a not negligible proportion featured no hepatic disease. Such a heterogeneous metabolic response to high-fat diet regimens in C57BL/6J mice was reported previously [28, 30, 34]. It was shown that genetic identical inbred C57BL/6J mice fed high-fat diet split in low and high metabolic responder, developing different phenotypes ranging from lean non-diabetic to lean diabetic and adipose diabetic [34]. With respect to liver morphology, different subgroups were identified [29, 30, 61]. Duval et al. showed that due to fat content in the used diet C57BL/6J mice develop different stages of liver disease. In this study mice were separated in low-fat low-responder featuring normal liver morphology, low-fat high-responder with benign hepatic lipid accumulation, high-fat low-responder, developing macrovesicular lipid droplets within hepatocytes, and high-fat high-responder, featuring steatosis in combination with ballooning and Mallory body formation as well as inflammatory cell infiltration [30]. Whether the astonishing heterogeneity in response to dietary regimens within genetically identical mice of an inbred strain originates from epigenetic mechanisms or is based on variation of gene copy numbers in the strain genome has still to be elucidated. Moreover, it remains uncertain why this heterogeneity is limited to C57BL/6 strain and does not occur in other inbred strains, e.g. 129Sv [27, 33, 34, 47, 61].

A higher fat content in the diet also induced obesity in CD-1 mice. Reduced circulating TG and elevated FFA as well as cholesterol in all dietary groups are pointing towards initial metabolic complications, but nevertheless no increase in TG storage in subcutaneous adipose tissue of HF fed CD-1 mice was observable. If more pronounced metabolic responses can be induced in CD-1 mice by prolonging the respective regimen duration has to be further elucidated. Nevertheless, from literature it is known that CD-1 mice, fed a high-fat diet for even 9 month, did not develop diabetes [28]. Thereby, insulin resistance as well as hyperinsulinemia are thought to influence hepatic free cholesterol content by activating low density lipoprotein receptor (LDLR) via SREBP-2 as well as hepatic cholesterol uptake. Hepatic free cholesterol content in turn, correlates with histological features of NAFLD [62, 63]. Unchanged hepatic free cholesterol content seen in all regimen treated CD-1 mice may thus be a consequence of probably missing hyperinsulinemia and an explanation for missing hepatic steatosis and inflammation apparent in this strain. If CD-1 mice are furthermore protected from high-fat diet-induced metabolic complications because they form white adipose tissue more efficiently than C57BL6/J mice, as speculated for BALB/c mice, or if resistance against diet-induced liver disease is based on additional mechanisms has to be further investigated [61].

129Sv mice showed no differences in weight increase between the dietary groups and weight only increased slightly in the respective groups. However, total adipose to body weight ratio was increased in nearly all dietary groups, due to enlarged subcutaneous adipose tissue extent, which might be a consequence of limited activity of 129Sv compared to C57BL/6 mice reported in literature [26]. TG storage in subcutaneous adipose tissue was also increased in HF fed 129Sv mice, indicating ongoing metabolic changes. Furthermore, metabolic abnormalities in all 129Sv dietary groups were evident from reduced serum TG concentration and hypercholesterolemia compared to untreated counterparts. Such metabolic changes due to high-fat diet feeding have been described in 129Sv mice before but where associated with weight increase and obesity [27, 47]. Another study by Almind et al. recognized only minor weight gain of 129Sv mice due to high-fat diet feeding; however, they did not examine changes in body composition [26]. Maybe obesity in the mentioned and in this study rather manifests as increased adipose to body weight ratio than increased weight gain. Furthermore, 129Sv mice were consistently prone to moderate diet-induced hepatic steatosis and inflammation and mice of all dietary groups developed these histopathological features of NAFLD/AFLD in the context of some metabolic changes.

Shown in literature and also here, most of the hepatic lipids are stored as TG during NAFLD/AFLD development, but also other lipids accumulate, e.g. free cholesterol, CE, CER and phospholipids. The accumulation of TG alone causes no hepatotoxic effects and may represent a hepatoprotective mechanism during FFA induced lipotoxicity [64]. This hypothesis is supported by previous studies in which a progression of NAFLD to NASH is accompanied by a decrease of diglyceride and TG levels [65] and cell culture experiments, showing that an overload of unsaturated FA leads to increased TG levels without apoptosis [66]. In this study, a significant increase in TG levels of all dietary regimen fed CD-1 and 129Sv mice was observed, pointing towards a more NAFLD/AFLD stage with initial inflammatory events rather than NASH/ASH. Additionally, also total hepatic cholesterol content was increased in all dietary regimen fed 129Sv mice, most reasonable due to an increase of CE in the respective groups. A similar observation in rodents was described before [67, 68] and is by some means contradictory to what was seen in humans with NAFLD and NASH, where free cholesterol is thought to play a pivotal role during the progression from NAFLD to NASH [69]. Free cholesterol was decreased in 129Sv mice due to ethanol feeding, but was increased due to a combination of high-fat diet and ethanol, maybe implicating ethanol as a potential trigger for progression of NAFLD. Also an inhere described increase in CER levels was observed in patients with NAFLD [70] as well as in mice with diet-induced NAFLD and is thought to be significant during inflammatory and apoptotic events, due to relevant signaling properties of CER [53, 65]. Although CER levels were also elevated in regimen treated CD-1 mice, this increase was more obvious in 129Sv mice. Higher CER content in livers of 129Sv mice might be a further explanation for higher susceptibility of this strain to develop diet-induced NAFLD, as CER are influencing not only inflammation but also hepatic TG accumulation and the insulin signaling pathway by e.g. altering translocation of IRS-1 [71–73].

Total FA were increased in livers of all dietary regimen treated 129Sv mice, which might promote cell dysfunction as well as apoptosis in hepatocytes. However, hepatic saturated FA were only elevated slightly in dietary regimen treated 129Sv mice, while MUFA showed a more prominent increase in the respective mice, pointing towards a SCD1 (stearoyl-coenzyme A desaturase 1) dependent detoxification of saturated FA through conversion to MUFA also in 129Sv mice [74]. A similar trend was observed for C57BL/6 mice fed with ethanol, which showed a decrease of hepatic saturated FA due to alcohol administration while unsaturated FA increased [53]. Hepatic lipid accumulation and inflammation in 129Sv mice may be promoted by an elongation of C16 to C18 FA, indicated by a slight increase of the C18 to C16 ratio [75, 76] and imbalances in FA desaturation, resulting in a more distinctive increase of the n-6 to n-3 ratio [77, 78]. Furthermore, Wang et al. showed that high-fat diet feeding in combination with CCl_4_ resulted in an accumulation of palmitic acid (C16:0) and stearic acid (C18:0) and a decrease in oleic acid (C18:1) and linoleic acid (C18:2) [53, 74]. Also in this study palmitic acid (C16:0) and stearic acid (C18:0) increased, but resulted only in significantly elevated levels by feeding a combination of high-fat and ethanol. These observations may indicate that high-fat diet feeding alone is not sufficient to induce these changes in hepatic FA profile. Contradictory to described by Wang et al., oleic acid (C18:1) as well as linoleic acid (C18:2) also increased in this study due high-fat diet feeding [74]. In mice as well as in humans with NAFLD and NASH, arachidonic acid (20:4n-6) is decreased, maybe because it is efficiently converted in pro-inflammatory molecules by cyclooxigenase enhancing hepatic inflammation and apoptosis [74]. This is contradictory to what was observed here, were arachidonic acid (20:4n-6) was similar or slightly increased due to dietary regimens.

In this study, we used HF, EtOH, and HF + EtOH feeding to also investigate differences in NAFLD/AFLD development associated with the respective dietary regimen. In C57BL/6 as well as in 129Sv mice, HF caused the most distinct effects on metabolic changes and hepatic disease followed by EtOH dietary regimen. Interestingly, feeding HF + EtOH was not associated with more severe features of NAFLD/AFLD. A similar result was described previously, where high-fat diet feeding resulted in more severe steatosis than ethanol feeding and a combined diet caused no further increase in hepatic TG accumulation or inflammatory response [31]. Nevertheless, a significant increase of free cholesterol in 129Sv mice fed with combined HF + EtOH as compared to HF or EtOH feeding alone, was obvious. However, whether HF + EtOH has more severe effects as herein described or if an overload of unsaturated FA, as indicated by the herein presented hepatic FA profile in 129Sv mice, protects against alcohol-induced liver disease and how these complex pathophysiological mechanisms are contributing to NAFLD/AFLD progression has to be further investigated.

Comparing C57BL/6 and 129Sv mice reveals similarities as well as substantial differences in their response to the herein used dietary regimens. C57BL/6 mice gained more weight than 129Sv mice both at HF and EtOH dietary regimen, whereat both strains showed increased subcutaneous adipose tissue due to all dietary regimens. This effect in high-fat diet-induced mouse models of NAFLD has been observed previously [30, 61] and represents a difference to human NAFLD, where the MS is associated with an expansion of visceral adipose tissue [2, 8]. As shown here and reported in literature, 129Sv mice exhibit higher serum TG levels and lower serum cholesterol concentrations compared to C57BL/6 mice on either dietary regimen, whereas FFA levels were similar in both strains [27, 47]. C57BL/6 mice exhibit a heterogeneous pattern of histopathological key features of NAFLD/AFLD, including enlarged hepatocytes, especially when fed HF. In contrast, 129Sv mice showed consistent steatosis and inflammation but no enlarged hepatocytes at all. Susceptibility of C57BL/6 mice and resistance of 129Sv mice to develop ballooned hepatocytes has also been shown by Hanada et al. [79]. The differences in hepatic lipid accumulation and subsequent hepatocyte ballooning as well as hepatic inflammation might be due to higher SREBP-1c (sterol regulatory element-binding protein-1c) and SCD1 levels in C57BL/6 mice compared to 129Sv mice, profoundly influencing TG turnover by induction of lipoprotein lipase as well as expression of PNPLA3 [27, 80, 81]. Nonetheless, genetically based differences in SREBP-1c and SCD1 levels do not explain why some of the C57BL/6 mice are prone to develop hepatic steatosis, while others are unaffected by high-fat diet feeding. In this context, other interesting studies showed that a SNP in PNPLA3 resulting in an I148M variant causes NAFLD in both humans and mice. The I148M mutant variant is through to cause accumulation of inactive PNPLA3 on lipid droplets and subsequent hepatic TG accumulation, observable under dietary conditions causing high hepatic insulin levels [80, 81]. Moreover, it was shown that SNPs in TM6SF2, influencing total cholesterol content, are also involved in hepatic lipid accumulation in humans [82]. These observations are pointing towards a pivotal role for SNPs in the susceptibility of individuals to develop NAFLD, which may be also relevant for mouse models of the disease. Additional studies at the genome level of frequently used WT mice are required to reveal the role for SNPs in modeling NAFLD/AFLD.

In conclusion, this study demonstrates that feeding Lieber DeCarli high-fat diet, Lieber DeCarli regular diet supplemented with ethanol, and a combination of Lieber DeCarli high-fat diet and ethanol induces histopathological features of hepatic steatosis and inflammation in C57BL/6 and 129Sv mice, but not in CD-1 mice. Thus, C57BL/6 and 129Sv are suitable to model NAFLD/AFLD-associated liver disease caused by different dietary regimens, whereat 129Sv exhibit a consistent response and are therefore more appropriate for studies with small number of mice. Liver disease more closely resemble those seen in human NAFLD/AFLD can be induced in C57BL/6 mice, but in experimental design it should be considered that not all mice will develop liver disease. In line with previous reports trying to model NAFLD in mice the genetic background could be identified to be critical [83]. Hence, for modeling NAFLD/AFLD genetic background of the used mouse strain and respective strain characteristics are highly relevant and should be taken into consideration when setting study protocols.

## Supporting Information

S1 Table Correlation of NAS and dietary regimen in C57BL/6, CD-1, and 129Sv mice. (DOCX)

S2 Table Medians and interquartile ranges of hepatic FA in 129Sv mice with respect to the different dietary regimens. (DOCX)
