# Supplementary material for: Susceptibility of Different Mouse Wild Type Strains to Develop Diet-Induced NAFLD/AFLD-Associated Liver Disease
Source: PLoS One. 2016 May 11;11(5):e0155163. doi: 10.1371/journal.pone.0155163 (PMC4863973; doi:10.1371/journal.pone.0155163)
Supplement: S1 Table — (DOCX) [file pone.0155163.s001.docx]

**S1 Table. Correlation of NAS and dietary regimen in CD-1, 129Sv, and C57BL/6 mice.**

| **untreated** | **HF** | **EtOH** | | | **HF + EtOH** | | |
| --- | --- | --- | --- | --- | --- | --- | --- |
|  | **7 weeks** | **12 weeks** | **14 weeks** | **16 weeks** | **5 weeks** | **7 weeks** | **9 weeks** |
| ns | C57BL/6 vs CD-1* | CD-1 vs 129Sv* | ns | ns | C57BL/6 vs 129Sv* | CD-1 vs 129Sv* | ns |

* differences were considered significant for *P* values of <0.05 by Kruskal-Wallis followed by Dunns test of selected pairs of columns

ns not significant
