# Supplementary material for: Susceptibility of Different Mouse Wild Type Strains to Develop Diet-Induced NAFLD/AFLD-Associated Liver Disease
Source: PLoS One. 2016 May 11;11(5):e0155163. doi: 10.1371/journal.pone.0155163 (PMC4863973; doi:10.1371/journal.pone.0155163)
Supplement: S2 Table — (DOCX) [file pone.0155163.s002.docx]

**S2 Table. Medians and interquartile ranges of hepatic FA in 129Sv mice with respect to the different dietary regimens.**

| **FA** | **untreated** | **HF** | **EtOH** | | | **HF + EtOH** | | |
| --- | --- | --- | --- | --- | --- | --- | --- | --- |
|  |  | **7 weeks** | **12 weeks** | **14 weeks** | **16 weeks** | **5 weeks** | **7 weeks** | **9 weeks** |
| **C14:0** | 0.0 (0.0 - 0.0) | 0.0 (0.0 - 0.0) | 0.3* (0.3 - 0.4) | 0.4 (0.1 - 0.4) | 0.2 (0.1 - 0.3) | 0.0 (0.0 - 0.4) | 0.2 (0.0 - 0.2) | 0.0 (0.0 - 0.1) |
| **C15:0** | 0.0 (0.0 - 0.0) | 0.0 (0.0 - 0.0) | 0.0 (0.0 - 0.1) | 0.0 (0.0 - 0.1) | 0.0 (0.0 - 0.0) | 0.0 (0.0 - 0.1) | 0.0 (0.0 - 0.1) | 0.0 (0.0 - 0.0) |
| **C16:0** | 31.8 (30.1 - 36.3) | 41.6 (38.9 - 44.7) | 50.8 (46.0 - 55.5) | 52.7 (47.8 - 56.1) | 56.9 (51.3 - 60.9) | 63.0 (39.3 - 73.1) | 76.7* (54.6 - 86.7) | 67.7* (60.8 - 76.5) |
| **C16:1 ω7cis** | 0.0 (0.0 - 0.8) | 0.2 (0.0 - 0.3) | 1.3 (1.0 - 1.5) | 1.5 (1.0 - 1.9) | 1.5* (1.3 - 2.3) | 0.4 (0.2 - 0.8) | 0.6 (0.5 - 0.8) | 0.4 (0.3 - 0.6) |
| **C16:1 ω7trans** | 0.0 (0.0 - 0.1) | 1.2 (0.7 - 1.3) | 2.9 (2.7 - 3.1) | 3.5* (2.7 - 3.7) | 3.6* (3.3 - 5.1) | 2.1 (1.1 - 3.2) | 3.7* (2.5 - 4.1) | 2.3 (2.0 - 3.1) |
| **C16:2 ω6,9allcis** | 0.0 (0.0 - 0.0) | 0.0 (0.0 - 0.0) | 0.0 (0.0 - 0.0) | 0.0 (0.0 - 0.0) | 0.0 (0.0 - 0.0) | 0.0 (0.0 - 0.2) | 0.0 (0.0 - 0.2) | 0.0 (0.0 - 0.0) |
| **C17:0** | 0.0 (0.0 - 0.0) | 0.2 (0.0 - 0.3) | 0.4* (0.4 - 0.6) | 0.5* (0.4 - 0.5) | 0.4 (0.4 - 0.5) | 0.3 (0.1 - 0.4) | 0.5* (0.3 - 0.6) | 0.3 (0.1 - 0.5) |
| **C18:0** | 30.6 (25.5 - 47.2) | 31.0 (26.6 - 34.1) | 32.7 (28.9 - 33.7) | 33.7 (31.6 - 35.5) | 32.9 (29.4 - 34.5) | 33.6 (26.3 - 44.6) | 32.8* (31.9 - 46.8) | 40.0* (36.3 - 43.8) |
| **C18:1 ω9cis** | 9.0 (7.7 - 14.3) | 79.5 (75.1 - 93.6) | 153.3 (110.8 - 174.7) | 155.1 (122.7 - 183.2) | 163.5 (140.7 - 207.9) | 158.3 (92.6 - 173.7) | 157.5* (154.8 - 229.8) | 178.7* (160.4 - 206.3) |
| **C18:1 ω9trans** | 1.4 (1.0 - 4.3) | 3.2 (3.1 - 3.5) | 1.3 (0.3 - 3.2) | 1.9 (0.3 - 5.0) | 0.8 (0.0 - 1.8) | 0.8 (0.5 - 5.5) | 0.1 (0.0 - 0.8) | 0.7 (0.1 - 4.3) |
| **C18:2 ω6,9allcis** | 19.0 (12.0 - 22.0) | 50.2* (46.5 - 84.2) | 21.7 (14.0 - 27.8) | 25.4 (18.3 - 32.0) | 24.9 (17.8 - 46.7) | 45.6 (39.1 - 72.2) | 66.5 (37.9 - 85.3) | 66.8* (52.3 - 87.5) |
| **C18:3 ω6,9,12allcis** | 0.0 (0.0 - 0.0) | 1.6 (1.1 - 1.8) | 0.5 (0.3 - 0.6) | 0.7 (0.5 - 0.9) | 0.9 (0.8 - 1.0) | 1.8* (1.0 - 2.6) | 1.9* (1.4 - 3.2) | 2.6* (2.3 - 3.1) |
| **C20:0** | 0.0 (0.0 - 0.2) | 1.4 (1.2 - 1.5) | 2.0 (1.1 - 2.6) | 1.2 (0.9 - 1.9) | 1.8 (1.6 - 2.0) | 1.9 (1.2 - 2.5) | 2.6* (1.8 - 3.1) | 2.8* (2.2 - 4.1) |
| **C20:1 ω7cis** | 0.0 (0.0 - 0.0) | 0.1 (0.1 - 0.2) | 0.6* (0.5 - 1.0) | 0.6* (0.4 - 0.7) | 1.0* (0.3 - 1.2) | 0.3 (0.1 - 0.8) | 0.5 (0.3 - 0.6) | 0.3 (0.2 - 0.4) |
| **C20:1 ω9cis** | 0.0 (0.0 - 0.0) | 3.8 (3.3 - 4.6) | 9.5* (8.7 - 11.0) | 9.7* (7.5 - 10.6) | 12.4* (9.9 - 14.4) | 6.3 (4.0 - 8.7) | 9.2 (6.8 - 9.6) | 7.3 (6.8 - 9.2) |
| **C20:2 ω6,9cisall** | 0.0 (0.0 - 0.5) | 2.5 (2.4 - 2.7) | 2.5 (2.5 - 2.6) | 2.5 (2.1 - 2.7) | 2.7 (2.2 - 3.4) | 5.0* (3.2 - 6.5) | 6.5* (5.6 - 7.9) | 5.0* (4.8 - 6.2) |
| **C20:2 ω9,12cisall** | 0.0 (0.0 - 0.0) | 0.1 (0.0 - 0.2) | 0.6* (0.6 - 0.7) | 0.7* (0.7 - 0.8) | 0.9* (0.6 - 1.0) | 0.4 (0.2 - 0.8) | 0.5 (0.2 - 0.6) | 0.3 (0.1 - 0.4) |
| **C20:3 ω6,9,12cisall** | 0.8 (0.0 - 2.2) | 5.2 (4.6 - 5.5) | 5.0 (4.7 - 5.5) | 5.6 (5.1 - 5.7) | 5.7 (5.3 - 7.1) | 9.1* (5.4 - 11.2) | 10.1* (8.5 - 12.8) | 8.9* (8.5 - 11.4) |
| **C20:3 ω9,12,15cisall** | 0.0 (0.0 - 0.0) | 0.3 (0.0 - 0.4) | 1.2* (1.0 - 1.2) | 1.4* (1.3 - 1.5) | 1.4* (1.1 - 1.8) | 0.5 (0.2 - 0.7) | 0.8 (0.6 - 0.8) | 0.7 (0.5 - 0.8) |
| **C20:4 ω6,9,12,15cisall** | 22.0 (18.8 - 30.3) | 29.3 (27.6 - 31.0) | 23.7 (23.2 - 25.5) | 23.9 (23.0 - 27.3) | 25.6 (24.9 - 26.5) | 27.0 (20.8 - 35.3) | 31.9 (28.4 - 38.2) | 34.0 (30.2 - 38.8) |
| **C22:0** | 0.8 (0.0 - 1.2) | 1.0 (0.9 - 1.0) | 0.9 (0.6 - 1.3) | 0.5 (0.4 - 0.9) | 0.7 (0.5 - 0.7) | 0.7 (0.6 - 1.1) | 0.7 (0.5 - 1.3) | 1.1 (0.8 - 1.6) |
| **C22:1 ω9cis** | 0.0 (0.0 - 0.0) | 0.2 (0.2 - 0.3) | 1.1* (0.7 - 1.5) | 0.6* (0.4 - 1.1) | 1.0* (0.9 - 1.3) | 0.3 (0.1 - 0.6) | 0.6 (0.4 - 0.9) | 0.9* (0.5 - 1.3) |
| **C22:2 ω6,9allcis** | 0.0 (0.0 - 0.0) | 0.0 (0.0 - 0.0) | 0.0 (0.0 - 0.1) | 0.0 (0.0 - 0.0) | 0.0 (0.0 - 0.1) | 0.0 (0.0 - 0.2) | 0.3 (0.0 - 0.4) | 0.3 (0.0 - 0.4) |
| **C22:3 ω6,9,12allcis** | 0.0 (0.0 - 0.0) | 0.0 (0.0 - 0.0) | 0.0 (0.0 - 0.0) | 0.0 (0.0 - 0.0) | 0.0 (0.0 - 0.0) | 0.0 (0.0 - 0.1) | 0.0 (0.0 - 0.1) | 0.0 (0.0 - 0.0) |
| **C22:4 ω6,9,12,15allcis** | 0.0 (0.0 - 0.4) | 2.6 (2.2 - 3.0) | 1.7 (1.6 - 2.2) | 2.1 (1.7 - 2.3) | 2.1 (2.1 - 3.0) | 4.1* (2.8 - 6.3) | 5.6* (4.5 - 6.8) | 5.1* (4.6 - 6.7) |
| **C22:5 ω3,6,9,12,15allcis** | 0.0 (0.0 - 0.3) | 0.4 (0.2 - 0.5) | 0.6 (0.5 - 0.7) | 0.6 (0.5 - 0.8) | 0.9* (0.7 - 1.1) | 0.6 (0.4 - 1.0) | 0.8* (0.6 - 1.0) | 0.6 (0.5 - 0.7) |
| **C22:5 ω6,9,12,15,18allcis** | 0.0 (0.0 - 0.9) | 4.9 (4.5 - 5.2) | 4.5 (4.0 - 5.0) | 4.7 (3.9 - 5.6) | 5.5 (4.5 - 6.2) | 6.7 (4.7 - 7.9) | 7.4* (6.1 - 11.0) | 9.0* (8.2 - 11.2) |
| **C22:6 ω3,6,9,12,15,18allcis** | 14.2 (6.3 - 14.8) | 8.9 (7.9 - 9.2) | 7.9 (7.6 - 9.3) | 8.4 (8.1 - 9.3) | 8.6 (6.8 - 8.8) | 8.3 (6.3 - 12.2) | 9.3 (8.3 - 10.9) | 9.6 (8.3 - 11.1) |
| **C23:0** | 0.0 (0.0 - 0.1) | 0.1 (0.0 - 0.2) | 0.2 (0.2 - 0.3) | 0.1 (0.0 - 0.2) | 0.1 (0.0 - 0.1) | 0.0 (0.0 - 0.2) | 0.0 (0.0 - 0.2) | 0.0 (0.0 - 0.0) |
| **C24:0** | 0.8 (0.0 - 0.9) | 0.6 (0.6 - 0.7) | 0.6 (0.6 - 0.9) | 0.5 (0.4 - 0.6) | 0.5 (0.4 - 0.6) | 0.5 (0.2 - 0.7) | 0.4 (0.4 - 0.7) | 0.5 (0.5 - 0.7) |
| **C24:1 ω9cis** | 0.3 (0.0 - 1.1) | 0.4 (0.3 - 0.6) | 1.0 (0.9 - 1.5) | 0.8 (0.6 - 1.0) | 1.1 (0.8 - 1.2) | 0.4 (0.2 - 0.7) | 0.4 (0.3 - 0.7) | 0.4 (0.3 - 0.6) |

* differences to untreated group were considered significant for *P* values of <0.05 by Kruskal-Wallis followed by Dunns test of selected pairs of columns
